# Massive cysticercosis and hydatidosis in a moose (*Alces alces*) from Poland

**DOI:** 10.1007/s00436-026-08671-9

**Published:** 2026-05-06

**Authors:** Katarzyna Filip-Hutsch, Jacek Karamon, Małgorzata Samorek-Pieróg, Zbigniew Wróblewski, Krzysztof Anusz, Anna M. Pyziel

**Affiliations:** 1 Department of Food Hygiene and Public Health Protection, Institute of Veterinary Medicine, Warsaw University of Life Sciences–SGGW, Nowoursynowska 159, 02-776 Warsaw, Poland; 2 Department of Parasitology, National Veterinary Research Institute, 57 Partyzantów Avenue, 24-100, Puławy, Poland; 3 Warmińsko-Mazurska Izba Lekarsko-Weterynaryjna, Gietkowska 1/1 LU, 10-170 Olsztyn, Poland; 4 Department of Public Health Protection and Animal Welfare, Institute of Veterinary Medicine, Faculty of Biological and Veterinary Sciences, Nicolaus Copernicus University in Toruń, 87-100 Toruń, Poland

**Keywords:** Metacestode, *Taenia hydatigena*, *Echinococcus canadensis* G8, Moose

## Abstract

*Taenia hydatigena* and *Echinococcus canadensis* G8 are taeniid cestodes maintained in transmission cycles involving carnivores and ungulates. This study reports a rare massive co-infection of both species in a free-ranging moose from northeastern Poland. A dissection of a four-year-old male moose, found in severe malnutrition in the area of Biebrza valley, northeastern Poland, revealed massive infection with Taeniidae larvae. Parasites were isolated and subjected to molecular identification. DNA was extracted, and fragments of the mitochondrial genes cytochrome c oxidase subunit 1 (*cox1*) and NADH dehydrogenase subunit 1 (*nad1*) were amplified by PCR, sequenced, and compared with reference sequences in the GenBank database. Molecular examination allowed identification of *T. hydatigena* and *E. canadensis* G8 metacestodes. Numerous *T. hydatigena* cysticerci were located in the omentum, mesentery, liver, and pleura, and hydatid cysts of *E. canadensis* G8 were found in the lungs and liver. Phylogenetic analysis showed identity with isolates from wild and domestic animals in Europe, Asia, and North America. The extensive parasite burden likely contributed to the animal’s poor condition and death. This study reveals a potential role of moose in the epidemiology of Taeniidae in Poland, indicating the environmental contamination by taeniid eggs and the need for assessing the transmission risk at the wildlife–domestic animal interface.

**Supplementary Information:**

The online version contains supplementary material available at 10.1007/s00436-026-08671-9.

## Introduction

Taeniidae is one of the most cosmopolitan tapeworm groups, with relevance to both animal and human health (Hoberg 2002; Nguyen et al. 2016). Adult tapeworms are located in the intestine of definitive hosts (carnivorous mammals), which become infected after ingestion of infective larvae. Larvae developes in the form of cysts in the peritoneal and pleural cavity, on the surface of visceral organs and in the nervous system of wide range of intermediate hosts (i.e. ruminants, wild boars, rodents, lagomorphs), causing cysticercosis, hydatidosis and coenurosis (Hoberg et al. 2000; Loos-Frank 2000; Scala and Varcasia 2006; Moro and Schantz 2008; Deplazes et al. 2019).

Some Taeniidae tapeworms, such as *Taenia hydatigena*, are common in domestic and farm animals, causing significant economic losses in livestock production (Perry and Sones 2007; Scala et al. 2015). However, the recent expansion of wildlife populations has increased the relevance of the sylvatic cycle in maintaining these parasites in the environment (Sgroi et al. 2019). Wild animals play an important role as reservoirs of tapeworm *T. hydatigena* with wolves as definitive hosts and wild ungulates, including wild boars, red deer, fallow deer and moose as intermediate hosts (Nguyen et al. 2016; Lesniak et al. 2008; Sgroi et al. 2020).

*Echinococcus canadensis* genotypes 8 (G8) and 10 (G10), earlier described as the ‘northern form’ of *E. granulosus* (Rausch 2003)*,* is a Taeniidae species, primarily transmitted in the sylvatic cycle with wolves and moose as principal hosts. Larvae of *E. canadensis* were reported in moose, wapiti, roe deer, and mule deer in Estonia, Russia, Canada, and the USA (Nakao et al. 2013; Cerda and Ballweber 2018; Priest et al. 2021; Laurimäe et al. 2023). in Europe, the occurrence of G8 is recorded south of the Baltic Sea, whereas G10 is associated with northern countries (Laurimäe et al. 2023).

Stable presence and high population density of wild ruminants seem to be crucial for the maintenance of the sylvatic life cycle of both *T. hydatigena* and *E. canadensis* (Joly and Messier 2004; Lesniak et al. 2008). Due to the dynamic increase of the moose (*Alces alces*) population in Poland, which is subject to a ban on moose hunting (Filip-Hutsch et al. 2021), this ruminant might play a significant role in the transmission of Taeniids in wildlife.

Herein, we report a rare case of an unusual massive infection of *T. hydatigena* and *E. canadensis* G8 in a moose in north eastern Poland.

## Materials and methods

A four-year-old male moose was found in a state of severe emaciation in Biebrza National Park, northeastern Poland, in November 2023. It was sedated, transported to the rehabilitation center, and died a few hours after waking from the anesthesia. Field dissection has been performed in the rehabilitation center of Biebrza National Park, immediately after the animal’s death by a qualified veterinarian, with the maintenance of hygienic rules, according to standard necropsy techniques and parasitological procedures. Parasite larvae were isolated from the organs and preserved in 70% ethanol in sterile tubes for further molecular analyses.

Genomic DNA was isolated from *T. hydatigena* metacestode using a NucleoSpin Tissue DNA extraction kit (Macherey–Nagel, Düren, Germany) according to the manufacturer’s protocol. The primers Thg452F (5′-TGCATTTAGCTGGTGCGTCAAGTA −3′) and Thg1326R (5′-ACAAACACGCCGGGGTAACC-3′) were used to partially amplify the mitochondrial cytochrome c oxidase subunit 1 gene (*co*x*1*). The PCR conditions followed Filip et al. (2019).

The PCR products were visualized on a 1.0% agarose gel (Promega) stained with ethidium bromide. Visualization was performed using ChemiDoc, MP Lab software (Imagine, Bio-Rad) (Fig. S1). The PCR amplicons were purified using a PCR Clean-Up Kit (Genoplast). The purified PCR products were sequenced in both directions by Genomed (Poland) and assembled into contigs using ContigExpress, Vector NTI Advance 11.0 (Invitrogen Life Technologies, USA). The derived sequences were submitted to GenBank/EMBL.

Extraction of DNA from the hydatid cysts was achieved using a QIAamp DNA Mini Kit (Qiagen, Hilden, Germany) following the manufacturer’s protocol. Isolates were analysed by amplification of the fragments of two mitochondrial genes: NADH dehydrogenase subunit 1 gene (*nad1*) and *cox1*. The first PCR assay described by Bowles and McManus (1993) and modified by Dybicz et al. (2018), with some minor modifications, was used to amplify a fragment of *nad1* with primers JB11 (5′-AGATTCGTAAGGGGCCTAATA-3′) and JB12 (5′-ACCACTAACTAATTCACTTTC-3′), producing an amplicon of approximately 500 bp. PCR reactions were performed using the Taq PCR Core Kit (Qiagen, Hilden, Germany). Each reaction was carried out in a final volume of 50 µL containing 5 µL of 10 × PCR buffer, 10 pmol of each primer, 200 pmol of each dNTP, 2 µL of 25 mM MgCl₂, 1 U of Taq DNA polymerase, 1 µL of template DNA, and 36.8 µL of nuclease-free water (Sigma-Aldrich, St. Louis, MO, USA). Amplification was performed with an initial denaturation step at 95 °C for 3 min, followed by 35 cycles consisting of denaturation at 95 °C for 60 s, annealing at 50 °C for 60 s, and extension at 72 °C for 60 s. The PCR program concluded with a final extension step at 72 °C for 5 min. The second PCR (amplified *cox1* fragment) was specified by Casulli et al. (2008). The PCR products were separated by horizontal electrophoresis in a 2% agarose gel stained by Simply Safe (EURx, Gdańsk, Poland) (Figs. S2 and S3). The selected PCR products were sequenced by standard Sanger sequencing at a commercial company (Genomed, Warsaw, Poland).. The received sequenced fragments of *cox1* and *nad1* of *E. canadensis* were edited and analysed in Geneious R11 (Kearse et al. 2012). Previously trimmed sequences were aligned according to ClustalW using the following parameters: gap-opening penalty 10 and gap-extension penalty 0.2. For the phylogenetic trees, a Tamura–Nei genetic distance model and the neighbour-joining method were used. One thousand nonparametric bootstrap inferences were performed. To estimate the phylogenetic position of the isolates, homologous mitochondrial DNA sequences from GenBank (Laurimäe et al. 2023, Dybicz et al. 2013; Yanagida et al. 2017) were retrieved and used in the analyses.

## Results

**Fig. 1:**
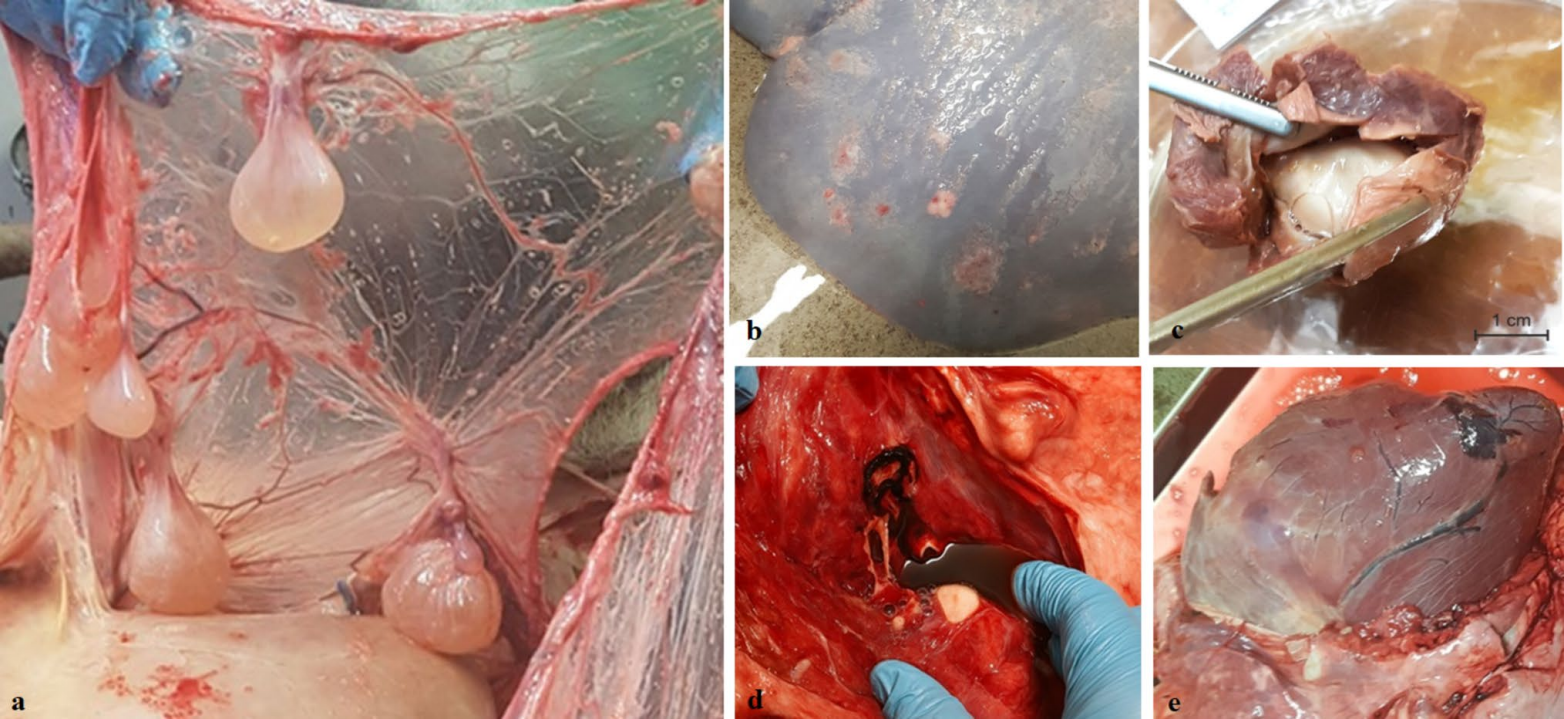
**a** *Taenia hydatigena* cysticerci in the omentum; **b** changes on the diaphragmatic surface of the liver; **c** hydatid cyst isolated from the lungs; **d** hydatid cyst on the cross-section of lungs; **e** gelatinous infiltrates on the pericardium

During the dissection, numerous cysts were visible in the abdominal cavity (Fig. 1a), covering the omentum and mesentery as well as the surface and parenchyma of the liver. Signs typical for liver degeneration have been observed (Fig. 1b). The pleural cavity was covered with numerous cysts. Lungs on the cross-section showed the presence of pea-sized white hydatid cysts containing fluid (with numerous protoscolices) (Fig. 1c) and numerous nodules of various consistencies (Fig. 1d). Features of interstitial pneumonia were observed. Enlargement of the heart’s right ventricle, gelatinous infiltrates on the pericardium, and coronary vessels filled with blood were also visible (Fig. 1e).

Isolated larvae have been identified as metacestodes of *T. hydatigena* and *E. canadensis* G8. Nucleotide sequences of the 787 bp of the *cox1* region for *T. hydatigena* (GenBank accession no: PP408289); 460 bp of *cox1* region and 519 bp of *nad*1 region for *E. canadensis* G8 (PV468348, PV471442) were obtained. Comparative analysis of the *T. hydatigena* sequences showed high nucleotide identity (99.5–99.8%) with homologous GenBank sequences from sheep and goats in Turkey (OQ317833), Ghana (MK945756), Algeria (MN175592), Nigeria (PV919957), and China (MT784878), roe deer in Poland (PP387599), and canines in China (MT784873).

**Fig. 2:**
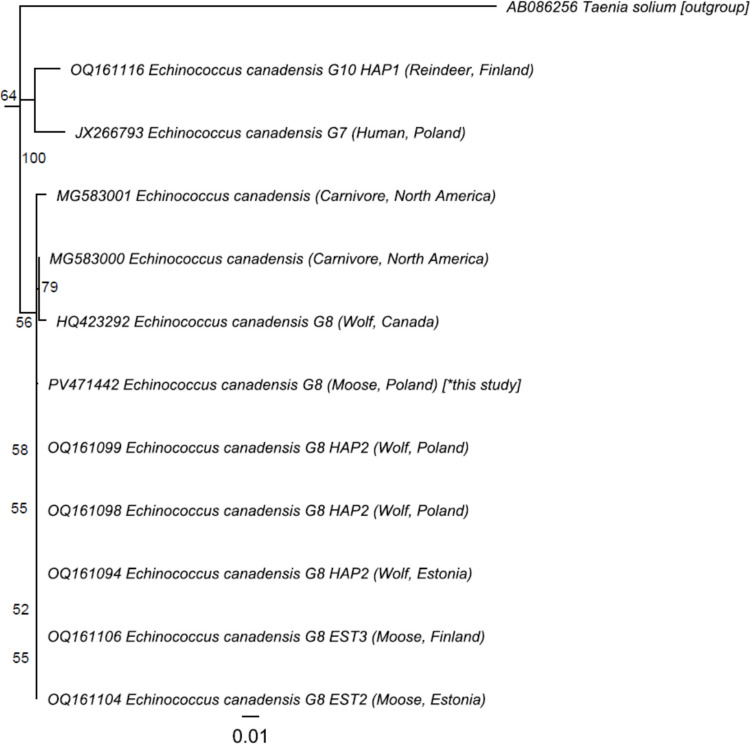
Phylogenetic tree of *Echinococcus canadensis* based on the nad1 gene (*– sequences of this study). Values on the tree nodes are bootstrap proportions (%)

**Fig. 3:**
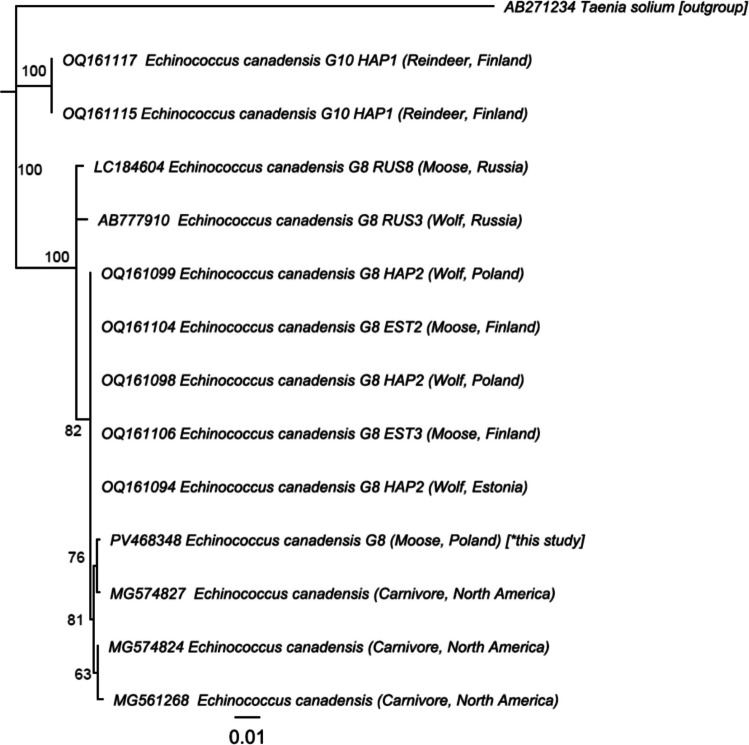
Phylogenetic tree of *Echinococcus canadensis* based on the *cox1* gene (*– sequences of this study). Values on the tree nodes are bootstrap proportions (%)

Comparison of *E. canadensis* sequences with others previously submitted in the GenBank database showed nearly full identity (99,5–99.8%) with the *E. canadensis* G8 sequences obtained from parasites found in moose and/or carnivore animals from Poland, Estonia, Finland, North America (Laurimäe et al. 2023, Dybicz et al. 2013; Yanagida et al. 2017) (Figs. 2 and 3).

## Discussion

Our study allowed us to identify larvae isolated from moose as *T. hydatigena* and *E. canadensis* G8 metacestodes. Although the exact number of parasites could not be determined, the observed infection of the moose was massive and probably contributed to the animal’s poor condition. Cysticercosis and hydatidosis are most often chronic and asymptomatic (Christodoulopoulos et al. 2008); however, clinical signs might vary according to the intensity of infection and localization of cysts (Sgroi et al. 2019; Romig and Wassermann 2024). Migration of numerous *T. hydatigena* larvae might cause hepatitis, peritonitis, and pneumonia, resulting in the death of the animal (Scala et al. 2014), which was previously reported in lambs (Scala et al. 2014, 2016; Edwards and Herbert 1980). A rare case of massive infection by *T. hydatigena* metacestodes was reported in a wild boar from Italy; however, no clinical sign was observed in the animal (Sgroi et al. 2019).

The principal reason for host mortality and morbidity in hydatidosis is considered the large size of cysts (Kern et al. 2017). In moose, as a typical intermediate host of *E. canadensis*, protoscolex production is much higher than in other ungulates (Schurer et al. 2013). Large lung cysts may reduce respiratory capacity and survival chances. Therefore, according to Joly and Mesier (2004), *E. canadensis* infection can increase the vulnerability of moose to predation.

The location of Taeniidae metacestodes was typical for both identified species. *T. hydatigena* larvae were presumably found in the omentum, mesentery, and on the surface of the liver, as well as in the pleura, which is considered less common (Pathak et al. 1982; Radfar et al. 2005). As for *E. canadensis* G8, the most common location is the lungs and liver, which was also observed in our study (Oksanen and Lavikainen 2015).

Infection with *T. hydatigena* metacestodes in moose was rarely described (Filip et al. 2019) despite being generally common in wild ungulates in Europe (Ngyuen et al. 2016; Lesniak et al. 2008; Sgroi et al. 2020). Similarly, there are only a few reports about *E. canadensis* G8 in moose (Laurimäe et al. 2023). However, the spread of this species in Europe has been poorly examined, as it was only recently described as a separate species (Romig et al. 2015). Limited data on the infection of moose with Taeniidae larvae might also result from the increasing but relatively low number of moose in Central Europe (Janík et al. 2021). Therefore, available records are fragmentary and do not necessarily reflect real parasitic prevalence.

The exact source of the massive moose infection in the present study remains unknown; however, it is possible that the animal ingested multiple Taeniidae eggs contaminating the soil (Sgroi et al. 2019). Few wild and domestic carnivore species are definitive hosts and potential reservoirs for tapeworms, causing cysticercosis and hydatidosis (Bandelj et al. 2022). *E. canadensis* G8 is primarily maintained in the sylvatic cycle with the principal definitive host wolf; however, spill-over to domestic dogs was also described (Lichtenwalner et al. 2014; Oksanen and Lavikainen 2015). In Poland, the presence of tapeworms belonging to the common *E. granulosus* s.l. complex in wolves was confirmed. Interestingly, in the work on the genetic diversity of *E. canadensis* (Laurimäe et al. 2023), two samples used by authors were tapeworms isolated from a Polish wolf (of unknown location) identified as *E. canadensis* G8 HAP2. The *cox1* and *nad1* sequence fragments from these samples were identical to the sequences obtained from cysts isolated from the moose described in this article. This may suggest wolves as a source of infection for this animal.

Although dogs are considered the primary definitive host of *T. hydatigena*, wolves may also play a role in the transmission of this parasite (Otranto et al. 2015; Lahmar et al. 2017). In studies conducted in south-eastern Poland, *T. hydatigena* prevalence in dogs was at a similarly low level (5.2%) (Karamon et al. 2019) as in wolves in the same area (8.1%) (Karamon et al. 2024). However, given that the dog population is many times larger than that of wolves, the number of eggs contaminating the environment through dog feces is also significantly higher. Nevertheless, massive moose infection, described in the present study, may indicate high contamination of the environment with Taeniidae eggs and may require further monitoring.

On the other hand, according to Rausch (2003), moose continue to accumulate *Echinococcus* metacestodes throughout their lives, which is also possible for *T. hydatigena* larvae (Filip et al. 2019). Therefore, it is possible that massive hydatidosis and cysticercosis in moose were a result of multiple infections over the years and not survival after a single intake of numerous cestode eggs.

The occurrence of identified parasites in the environment might have implications for wildlife health and the transmission of infectious diseases. Interactions between domestic and wild animals for both Taeniidae species are possible and might be favored by improper disposal of raw offals while hunting, stray dogs, and home slaughtering (Sgroi et al. 2019; Oksanen and Lavikainen 2015). Determining the range of both Taeniidae species and the occurrence of tapeworms in wild and domestic animals in the studied area is crucial to determine the risk of infection.

## Supplementary Information

Below is the link to the electronic supplementary material.

Supplementary file1 (DOC 4199 kb)

## Data Availability

The data that support the findings of this study are available from the corresponding author upon reasonable request.

## References

[CR1] Bandelj P, Blagus R, Vengušt G, Žele Vengušt D (2022) Wild carnivore survey of *Echinococcus* species in Slovenia. Animals 12:2223. 10.3390/ani12172223 36077943 10.3390/ani12172223 PMC9454715

[CR2] Bowles J, McManus DP (1993) NADH dehydrogenase-1 gene-sequences compared for species and strains of the genus *Echinococcus*. Int J Parasitol 23:969–972. 10.1016/0020-7519(93)90065-7 8106191 10.1016/0020-7519(93)90065-7

[CR3] Casulli A, Manfredi MT, La Rosa G, Di Cerbo AR, Genchi C, Pozio E (2008) *Echinococcus ortleppi* and *E. granulosus* G1, G2 and G3 genotypes in Italian bovines. Vet Parasitol 155:168–172. 10.1016/j.vetpar.2008.04.004 18514422 10.1016/j.vetpar.2008.04.004

[CR4] Cerda JR, Ballweber LR (2018) Confirmation of *Echinococcus canadensis* G8 and G10 in Idaho gray wolves (*Canis lupus*) and cervids. J Wildl Dis 54:403–405. 10.7589/2017-05-119 29369720 10.7589/2017-05-119

[CR5] Christodoulopoulos G, Theodoropoulos G, Petrakos G (2008) Epidemiological survey of cestode-larva disease in Greek sheep flocks. Vet Parasitol 153:368–373. 10.1016/j.vetpar.2008.02.002 18346853 10.1016/j.vetpar.2008.02.002

[CR6] Deplazes P, Eichenberger RM, Grimm F (2019) Wildlife-transmitted *Taenia* and *Versteria* cysticercosis and coenurosis in humans and other primates. Int J Parasitol Parasites Wildl 9:342–358 31338294 10.1016/j.ijppaw.2019.03.013 PMC6626850

[CR7] Dybicz M, Gierczak A, Dabrowska J, Rdzanek L, Michalowicz B (2013) Molecular diagnosis of cystic echinococcosis in humans from central Poland. Parasitol Int 62:364–367. 10.1016/j.parint.2013.03.005 23535071 10.1016/j.parint.2013.03.005

[CR8] Dybicz M, Borkowski PK, Padzik M, Baltaza W, Chomicz L (2018) Molecular determination of suspected alveolar echinococcosis requiring surgical treatment in human cases from Poland. Ann Parasitol 64:339–342 30726664

[CR9] Edwards GT, Herbert IV (1980) The course of *Taenia hydatigena* infections in growing pigs and lambs: clinical signs and post-mortem examination. Br Vet J 136:256–264. 10.1016/s0007-1935(17)32290-x 7388589 10.1016/s0007-1935(17)32290-x

[CR10] Filip KJ, Pyziel AM, Jeżewski W, Myczka AW, Demiaszkiewicz AW, Laskowski Z (2019) First molecular identification of *Taenia hydatigena* in wild ungulates in Poland. EcoHealth 16:161–170. 10.1007/s10393-019-01392-9 30673904 10.1007/s10393-019-01392-9 PMC6430758

[CR11] Filip-Hutsch K, Czopowicz M, Barc A, Demiaszkiewicz AW (2021) Gastrointestinal helminths of a European moose population in Poland. Pathogens 10:456. 10.3390/pathogens10040456 33920333 10.3390/pathogens10040456 PMC8070461

[CR12] Hoberg EP (2002) *Taenia* tapeworms: their biology, evolution and socioeconomic significance. Microbes Infect 4:859–866. 10.1016/s1286-4579(02)01606-4 12270733 10.1016/s1286-4579(02)01606-4

[CR13] Hoberg EP, Jones A, Rausch RL, Eom KS, Gardner SL (2000) A phylogenetic hypothesis for species of the genus *Taenia* (Eucestoda: Taeniidae). J Parasitol 86:89–98. 10.1645/0022-3395(2000)086[0089:APHFSO]2.0.CO;2 10701570 10.1645/0022-3395(2000)086[0089:APHFSO]2.0.CO;2

[CR14] Janík T, Peters W, Šálek M, Romportl D, Jirků M, Engleder T, Ernst M, Neudert J, Heurich M (2021) The declining occurrence of moose (*Alces alces*) at the southernmost edge of its range raise conservation concerns. Ecol Evol 11:5468–5483. 10.1002/ece3.7441 34026021 10.1002/ece3.7441 PMC8131793

[CR15] Joly DO, Messier F (2004) The distribution of *Echinococcus granulosus* in moose: evidence for parasite-induced vulnerability to predation by wolves? Oecologia 140:586–590. 10.1007/s00442-004-1633-0 15232731 10.1007/s00442-004-1633-0

[CR16] Karamon J, Sroka J, Dabrowska J, Bilska-Zajac E, Zdybel J, Kochanowski M, Rozycki M, Cencek T (2019) First report of *Echinococcus multilocularis* in cats in Poland: a monitoring study in cats and dogs from a rural area and animal shelter in a highly endemic region. Parasit Vectors 12:313. 10.1186/s13071-019-3573-x 31234884 10.1186/s13071-019-3573-x PMC6591820

[CR17] Karamon J, Samorek-Pieróg M, Bilska-Zajac E, Korpysa-Dzirba W, Sroka J, Zdybel J, Cencek T (2024) The grey wolf (*Canis lupus*) as a host of and other helminths - a new zoonotic threat in Poland. J Vet Res 68:539–549. 10.2478/jvetres-2024-0060 39776693 10.2478/jvetres-2024-0060 PMC11702251

[CR18] Kearse M, Moir R, Wilson A, Stones-Havas S, Cheung M, Sturrock S, Buxton S, Cooper A, Markowitz S, Duran C, 10.1093/bioinformatics/bts199 PMC3371832 22543367

[CR19] Kern P, da Silva AM, Akhan O, Müllhaupt B, Vizcaychipi KA, Budke C, Vuitton DA (2017) The echinococcoses: diagnosis, clinical management and burden of disease. Adv Parasitol 96:259–369. 10.1016/bs.apar.2016.09.006 28212790 10.1016/bs.apar.2016.09.006

[CR20] Lahmar S, Arfa I, Ben Othmen S et al (2017) Intestinal helminths of stray dogs from Tunisia with special reference to zoonotic infections. Parasitol Open 3:e18. 10.1017/pao.2017.21

[CR21] Laurimäe T, Kinkar L, Moks E, Bagrade G, Saarma U (2023) Exploring the genetic diversity of genotypes G8 and G10 of the *Echinococcus canadensis* cluster in Europe based on complete mitochondrial genomes (13 550–13 552 bp). Parasitology 150:631–637. 10.1017/s0031182023000331 37005069 10.1017/S0031182023000331 PMC10260296

[CR22] Lesniak I, Heckmann I, Heitlinger E, Szentiks CA, Nowak C, Harms V, Jarausch A, Reinhardt I, Kluth G, Hofer H, Letkova V, Lazar P, Soroka J, Goldova M, Urlik J (2008) Epizootiology of game cervid cysticercosis. Nat Croat 17:311–318

[CR23] Lichtenwalner A, Adhikari N, Kantar L, Jenkins E, Schurer J (2014) *Echinococcus granulosus* genotype G8 in Maine moose (*Alces alces*). Alces: J Devoted Biol Manag Moose 50:27–33

[CR24] Loos-Frank B (2000) An up-date of Verster’s (1969) `Taxonomic revision of the genus *Taenia* Linnaeus’ (Cestoda) in table format. Syst Parasitol 45:155–183. 10.1023/a:1006219625792 10768761 10.1023/a:1006219625792

[CR25] Moro P, Schantz PM (2008) Echinococcosis: a review. Int J Infect Dis 13:125–133. 10.1016/j.ijid.2008.03.037 18938096 10.1016/j.ijid.2008.03.037

[CR26] Nakao M, Yanagida T, Konyaev S, Lavikainen A, Odnokurtsev VA, Zaikov VA, Ito A (2013) Mitochondrial phylogeny of the genus *Echinococcus* (Cestoda: Taeniidae) with emphasis on relationships among *Echinococcus canadensis* genotypes. Parasitology 140:1625–1636. 10.1017/s0031182013000565 23731519 10.1017/S0031182013000565

[CR27] Nguyen MT, Gabriël S, Abatih EN, Dorny P (2016) A systematic review on the global occurrence of *Taenia hydatigena* in pigs and cattle. Vet Parasitol 226:97–103. 10.1016/j.vetpar.2016.06.034 27514893 10.1016/j.vetpar.2016.06.034

[CR28] Oksanen A, Lavikainen A (2015) *Echinococcus canadensis* transmission in the North. Vet Parasitol 213:182–186. 10.1016/j.vetpar.2015.07.033 26264249 10.1016/j.vetpar.2015.07.033

[CR29] Otranto D, Cantacessi C, Dantas-Torres F, Brianti E, Pfeffer M et al (2015) The role of wild canids and felids in spreading parasites to dogs and cats in Europe. Part II: Helminths and arthropods. Vet Parasitol. 10.1016/j.vetpar.2015.04.020 26049678 10.1016/j.vetpar.2015.04.020

[CR30] Pathak KML, Gaur SNS, Sharma SN (1982) The pathology of *Cysticercus tenuicollis* infection in goats. Vet Parasitol 11:131–139 6891847 10.1016/0304-4017(82)90035-8

[CR31] Perry B, Sones K (2007) Poverty reduction through animal health. Science 315:333–334. 10.1126/science.1138614 17234933 10.1126/science.1138614

[CR32] Priest JM, McRuer DL, Stewart DT, Boudreau M, Power JWB, Conboy G, Jenkins EJ, Kolapo TU, Shutler D (2021) New geographic records for *Echinococcus canadensis* in coyotes and moose from Nova Scotia, Canada. Int J Parasitol- Parasites Wildl 16:285–288. 10.1016/j.ijppaw.2021.11.004 34917469 10.1016/j.ijppaw.2021.11.004 PMC8646049

[CR33] Radfar MH, Tajalli S, Jalalzadeh M (2005) Prevalence and morphological characterization of *Cysticercus tenuicollis* (*Taenia hydatigena* cysticerci) from sheep and goats in Iran. Veterinarski Arhiv 75(6):469–476

[CR34] Rausch RL (2003) Cystic echinococcosis in the arctic and sub-arctic. Parasitology 127(Suppl. l):S73–S85. 10.1017/s0031182003003664 15027606 10.1017/s0031182003003664

[CR35] Romig T, Wassermann M (2024) *Echinococcus* species in wildlife. Int J Parasitol Parasites Wildl 23:100913. 10.1016/j.ijppaw.2024.100913 38405672 10.1016/j.ijppaw.2024.100913 PMC10884515

[CR36] Romig T, Ebi D, Wassermann M (2015) Taxonomy and molecular epidemiology of *Echinococcus granulosus* sensu lato. Vet Parasitol 213:76–84. 10.1016/j.vetpar.2015.07.035 26264250 10.1016/j.vetpar.2015.07.035

[CR37] Scala A, Varcasia A (2006) Updates on morphobiology, epidemiology and molecular characterization of coenurosis in sheep. Parassitologia 48:61–63 16881398

[CR38] Scala A, Urrai G, Varcasia A, Nicolussi P, Mulas M, Goddi L, Pipia AP, Sanna G, Genchi M, Bandino E (2014) Acute visceral cysticercosis by *Taenia hydatigena* in lambs and treatment with praziquantel. J Helminthol 14:1–4. 10.1017/S0022149X14000601 10.1017/S0022149X14000601 25120032

[CR39] Scala A, Pipia AP, Dore F, Sanna G, Tamponi C, Marrosu R, Bandino E, Carmona C, Boufana B, Varcasia A (2015) Epidemiological updates and economic losses due to *Taenia hydatigena* in sheep from Sardinia, Italy. Parasitol Res 114:3137–3143. 10.1007/s00436-015-4532-x 25968992 10.1007/s00436-015-4532-x

[CR40] Scala A, Urrai G, Varcasia A, Nicolussi P, Mulas M, Goddi L, Pipia AP, Sanna G, Genchi M, Bandino E (2016) Acute visceral cysticercosis by *Taenia hydatigena* in lambs and treatment with praziquantel. J Helminthol 90:113–116. 10.1017/S0022149X14000601 25120032 10.1017/S0022149X14000601

[CR41] Schurer J, Shury T, Leighton F, Jenkins E (2013) Surveillance for *Echinococcus canadensis* genotypes in Canadian ungulates. Int J Parasitol Parasites Wildl 2:97–101. 10.1016/j.ijppaw.2013.02.004 24533321 10.1016/j.ijppaw.2013.02.004 PMC3862526

[CR42] Sgroi G, Varcasia A, Dessi G, D’Alessio N, Tamponi C, Saarma U, Laurimäe T, Kinkar L, Santoro M, Caputo V, Sgroi G, Varcasia A, Dessì G, D’Alessio N, Pacifico L, Buono F, Neola B, Fusco G, Santoro M, Toscano V, Fioretti A, Veneziano V (2019) Massive *Taenia hydatigena* cysticercosis in a wild boar (*Sus scrofa*) from Italy. Acta Parasitol 64:938–941. 10.2478/s11686-019-00110-3 31444647 10.2478/s11686-019-00110-3

[CR43] Sgroi G, Varcasia A, D’Alessio N, Varuzza P, Buono F, Amoroso MG, Boufana B, Otranto D, Fioretti A, Veneziano V (2020) *Taenia hydatigena* cysticercosis in wild boar (*Sus scrofa*) from southern Italy: an epidemiological and molecular survey. Parasitology 147:1636–1642. 10.1017/S0031182020001559 32829716 10.1017/S0031182020001559 PMC7708990

[CR44] Yanagida T, Lavikainen A, Hoberg EP, Konyaev S, Ito A, Sato MO, Zaikov VA, Beckmen K, Nakao M (2017) Specific status of *Echinococcus canadensis* (Cestoda: Taeniidae) inferred from nuclear and mitochondrial gene sequences. Int J Parasitol 47:971–979. 10.1016/j.ijpara.2017.07.001 28797792 10.1016/j.ijpara.2017.07.001

