# Supplementary material for: Massive cysticercosis and hydatidosis in a moose (Alces alces) from Poland
Source: Parasitol Res. 2026 May 6;125(1):75. doi: 10.1007/s00436-026-08671-9 (PMC13319169; doi:10.1007/s00436-026-08671-9)
Supplement: Supplementary file 1 — Supplementary file1 (DOC 4199 kb) [file 436_2026_8671_MOESM1_ESM.doc]

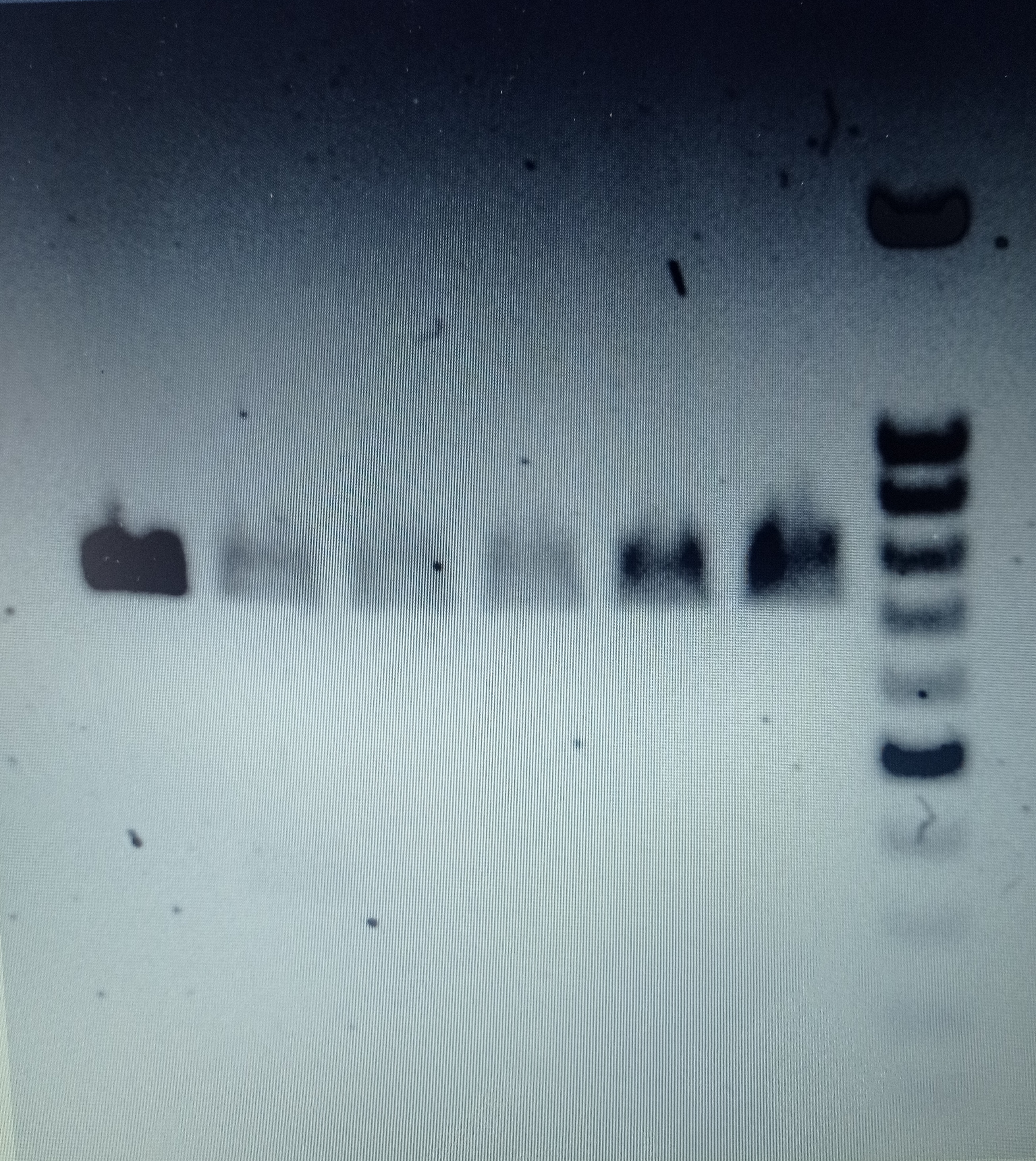


Fig. S1. Image of electrophoresis gel of cox1 fragment of *T*. *hydatigena.*


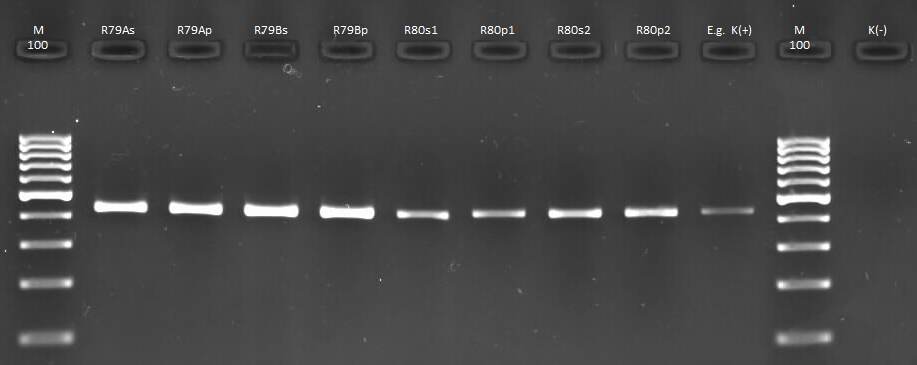


Fig. S2. Image of electrophoresis gel of *cox1* fragment of *E. canadensis* G8 (R80)


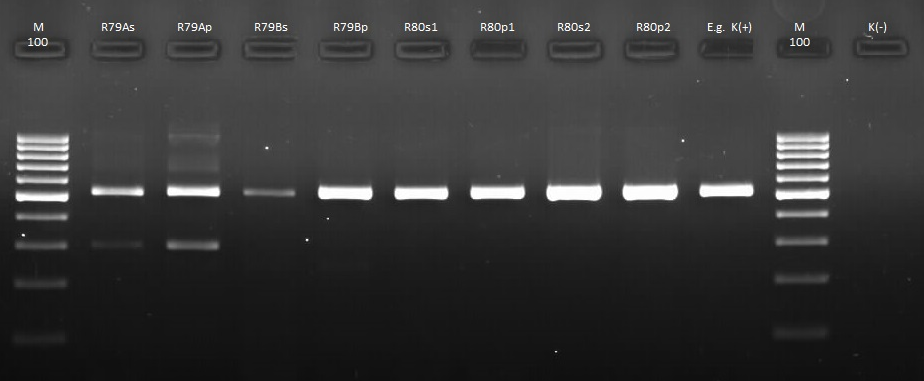


Fig. S3 Image of electrophoresis gel of *nad1* fragment of *E. canadensis* G8 (R80).
